# The Much-Abused Title of Doctor of Medicine

**Published:** 1911-03

**Authors:** 


### Abstracts and Selections.

### The Much-Abused Title of Doctor of Medicine.

In the good old days, as the conventional phrase runs, when simplicity marked our medical sociology, the title of doctor preceding a name, be it never so commonplace, meant a distinction that could not be easily misconstrued. True, even in those times of uncomplicated machinery, a number of disreputables assumed honors which did not rightly belong to them, but, though in certain communities our lax laws allowed this dereliction to wax strong, the acumen of the fairly intelligent stood them in good stead in the matter of differentiating between the real and the bogus article. In short, a quack bore the stigma of his class and, being under the burden of an ostracism that meant considerable cold-shouldering on the part of those whose money would have been thrice welcome, he resorted to the only medium which was open to him through which to announce his virtues to a stubborn public—the daily press. By doing this he declared his complete segregation from those who held diplomas that were worthy of the name; he made money, it is true, but his glory did not last; for being an ordinary human being, in most cases, with a too evident desire to reap the richest harvest, he played to a contingent in a community that wants something in return for its money, though in the beginning it may be dazzled by bombast. And so before long, the fat, gilt lettering, which had beautified the time-worn brick front of the building which housed the quack—old buildings were always preferred, perhaps, because quackery is so much older than real medical science—vanished, as if by magic; the cobwebs returned to their former haunts; and "Ichabod, thy glory is departed" was the message to be garnered by the wary. Strange, as it may seem, the meretricious irruptions of this special form of quackery into communities was lightly thought of; perhaps, too lightly, but then it must be stated in extenuation of what was a too benign attitude that we were too light-hearted and too childish a people at that time to recognize the value of the inherent beauties and advantages and stringencies of the laws which are with us at present.

With the passing of the sort of quack, whom we have attempted to describe, the qualified practitioner grew more hopeful; and, though he should have known that one evil begets another, he gave but small thought of what was in store for him. This mental attitude was not due to any romantic notions as to what this very real world spelt for him, but rather to the fact that with the obstreperous and ubiquitous quack out of the way, there was no need to fear that any serious molestation could arrive from other sources. In short, he believed that the title, which he bore with considerable pride, would not again be tarnished by any one whose practices might be construed, by the unthinking part of the laity, as similar to his own. Complaisance such as this was bound to be disturbed, especially in the medical profession, the door of which for some unexplainable reason is always too greatly ajar; and before many days elapsed the blazed trail, which had been deserted since the quack had been unrooted, was again invaded by those whose ways were gentle when compared with the sensational outpourings of their predecessors, but whose obscure workings and general lack of medical education nevertheless made them an undeniable menace to the fair name of doctor.

Now, since this is the case, what recourse has the reputable physician when he hears that the irregulars and opticians, having taken their courses of studies, are just as much entitled to the much-coveted prefix as he is, though they may have pored over their peculiar studies only for a month or a year—and a very short one at that—while for him to arrive at his goal meant achievement by the sweat of his brow? True, he will be told by his fellowmen in the ranks of medicine that his superiority to the "others" is so apparent that he need fear no harm from invidious comparisons, even when these are made by people who ought to know better; that the laws are ever and ever increasing in stringency; and that before long—what a convenient phrase "before long" is!—the wrongs, which now grate on his nerves, will be righted. But these words, which fairly drip with the optimism that one would apply to a recalcitrant child that grows restive under imaginary wrongs, are really wide of the mark, not only because their reiteration has robbed them of their pristine strength, but because the initiated know that they are offered in the spirit of a makeshift. They are sure of this because on all sides there are evidences that the improvements so much talked about are not apparent, even to the most observant, and that the supineness which permeates the rank and file of humanity continues to make light of a differentiation between the holder of a diploma from a medical college of standing and the offspring of a frayed alma mater, so long as the cabalistic prefix declares the latter to be one of the elect.

After studying the conditions, as they exist in all our large cities, are we wrong in saying that it would be advisable for the qualified practitioners to take the matter in their own hands, if they hope for a fruition of the desire to elevate the title of doctor of medicine to the heights upon which it rested until it was dragged through the mire by the unscrupulous? Would it be futile to evolve a plan such as this: All qualified practitioners be compelled to write "doctor of medicine," i. e., Dr. med, in front of their names?—a compulsion that should show no laxity, since its object is not only to instruct the people at large, but prevent the osteopath, the optician and others too numerous to mention, from arrogating to themselves an honor that, on account of its bastardy, is today an exceedingly commonplace designation. Now this may sound Utopian, but it is done in other countries—namely, in the German-speaking ones, where systematization is a cult that it would be well for us to follow. Of course, there is no denying that this would be a slavish imitation of something that is foreign —hence, despicable; but despite this apparently insuperable drawback, and not overlooking the awkwardness of the more elaborate designation, would not the results counteract these and other objections? We think they would, for the reason that almost immediately the line of demarcation would plainly show who is the doctor of medicine and who is the doctor masquerading in soiled and tattered garments that must be cautionsly arranged to hide his igonrances.—*Interstate Medical Journal.*

